# Closed-Form Expression for the Symbol Error Probability in Full-Duplex Spatial Modulation Relay System and Its Application in Optimal Power Allocation

**DOI:** 10.3390/s19245390

**Published:** 2019-12-06

**Authors:** Le Van Nguyen, Ba Cao Nguyen, Xuan Nam Tran, Le The Dung

**Affiliations:** 1 Advanced Wireless Communications Group, Le Quy Don Technical University, Hanoi 11917, Vietnam; nguyenlevan2211@gmail.com (L.V.N.); bacao.sqtt@gmail.com (B.C.N.); namtx@mta.edu.vn (X.N.T.); 2 Division of Computational Physics, Institute for Computational Science, Ton Duc Thang University, Ho Chi Minh City 758307, Vietnam; 3 Faculty of Electrical and Electronics Engineering, Ton Duc Thang University, Ho Chi Minh City 758307, Vietnam

**Keywords:** spatial modulation, multiple-input multiple-output, full-duplex, self-interference cancellation, symbol error probability

## Abstract

Full-duplex (FD) communication and spatial modulation (SM) are two promising techniques to achieve high spectral efficiency. Recent works in the literature have investigated the possibility of combining the FD mode with SM in the relay system to benefit their advantages. In this paper, we analyze the performance of the FD-SM decode-and-forward (DF) relay system and derive the closed-form expression for the symbol error probability (SEP). To tackle the residual self-interference (RSI) due to the FD mode at the relay, we propose a simple yet effective power allocation algorithm to compensate for the RSI impact and improve the system SEP performance. Both numerical and simulation results confirm the accuracy of the derived SEP expression and the efficacy of the proposed optimal power allocation.

## 1. Introduction

Full-duplex (FD) communication and spatial modulation (SM) are two promising techniques to increase the spectral efficiency of wireless systems [1,2,3]. Theoretically, an FD communication system can double the spectral efficiency as its transceivers can transmit and receive signals at the same time and on the same frequency [4,5]. Besides increasing the channel capacity, the FD communication systems can also reduce the end-to-end and feedback delay, improve network security and solve the hidden terminal problem. Therefore, the FD mode has found its applications in various wireless systems such as sensor networks, massive MIMO, relaying networks and possibly future wireless networks such as the fifth-generation (5G) and beyond. Unfortunately, the residual self-interference (RSI) due to imperfect self-interference cancellation (SIC) limits the capacity and performance of the FD communication systems [6]. In the literature, numerous solutions such as relay selection scheme and adaptive transmission [7,8] and optimal power allocation [9,10,11] were proposed to reduce the impact of the RSI and improve the performance of the FD relay communication system. These solutions significantly improved the capacity, outage performance and energy efficiency of the single-input single-output (SISO) systems.

Meanwhile, the multiple-input multiple-output (MIMO) transmission is another solution that can also achieve high capacity and better performance over the SISO systems [12]. However, the hardware of the MIMO system is more complex, that is, more radio frequency (RF) chains, as both transmitter and receiver user multiple antennas. The MIMO receiver also requires a high-complexity detector to deal with the inter-channel interference (ICI) due to simultaneous transmissions on the same frequency from different antennas. In that context, SM can tackle these issues correctly as it activates only one transmit antenna for transmission and requires only low-complexity iterative maximal-ratio combining (MRC) at the receiver for signal estimation [12,13,14,15]. Therefore, the combined SM and FD system achieves higher spectral efficiency while reducing the complexity requirement at the transceiver, particularly of the relay node.

In the literature, using FD relay in SM systems has attracted great interest because this integrated FD-SM system has the advantages of both spectral efficiency improvement and performance enhancement. Numerous works focused on analyzing the system performance in terms of the outage probability (OP) [2], bit error rate (BER) [3], average symbol error probability (SEP), ergodic capacity [16] and average BER [17,18]. Specifically, in Reference [2], the lower and upper bounds of the OP of the SM-MIMO system with decode-and-forward (DF) FD relay were derived over cascaded α−μ fading channels. It also demonstrated that the RSI had a strong impact on the OP performance of the system. The work in Reference [3] considered the SM-MIMO system with amplify-and-forward (AF) FD/half-duplex (HD) relaying. It successfully derived a new unified tight upper-bound for the system BER. The results of the paper indicate that the SM-MIMO-FD relay system can improve the BER and the spectral efficiency if suitable SIC techniques are applied. Under the same assumption of the RSI, References [16,17,18] investigated the SM-MIMO-FD relay systems which can exploit the benefits of the FD transmission mode. The approximate expressions of SEP ([16]) and BER ([17,18]) were also derived for performance evaluation.

Although the previous works conducted various performance analyses, their results were limited to either upper and lower bounds or approximate expressions but not the exact closed-form expressions of SEP and BER. Therefore, it is required to have exact mathematical expressions for the performance evaluation rather than the upper bound or approximate ones for better understanding the system behaviors. Moreover, since the FD mode significantly degrades the system performance, besides effective SIC techniques, there should be other solutions such as power allocation to compensate for this degradation. Motivated by the previous works, in this paper, we aim to derive the exact closed-form expression of the SEP of the SM-FD relay system with DF protocol applied at the relay to enhance the system performance. Based on the derived expression of SEP, we can determine the optimal power allocation for the FD relay to reduce the RSI impact on the SEP performance. So far, this is the first work that successfully derives the exact closed-form expression of SEP and use it for optimizing the power allocation for SM-MIMO-FD relay systems. The main contributions of this paper can be summarized as follows:

- We analyze the SM-MIMO-FD relay system where SM is used at the source and the relay nodes under the impact of the RSI caused by the imperfect SIC. Unlike previous works, we derive the exact closed-form expression of SEP for the system over the Rayleigh fading channel.
- We propose an algorithm to calculate the optimal transmission power of the FD relay. Based on this algorithm, we obtain the optimal power allocation for the considered system. The proposed optimal transmission power algorithm significantly improves the SEP performance, especially in the low SNR region. Additionally, using the derived expression of SEP, we can also examine the influences of the number of transmitting/receiving antennas and the RSI on the system performance in the case with and without optimal power allocation.

The rest of this paper is organized as follows. Section 2 presents the system model. Section 3 provides the detailed derivations of the closed-form expression of SEP. The optimal power allocation algorithm for the FD relay is developed in Section 3. Section 4 presents the analytical and simulation results for performance evaluation. Finally, Section 5 concludes the paper.

## 2. System Model

**Figure 1:**
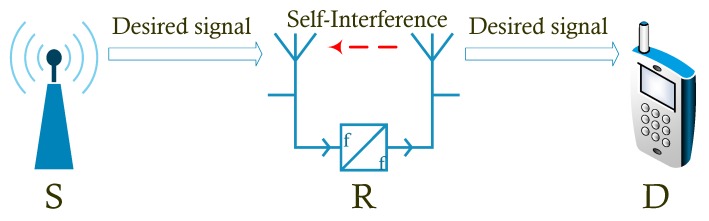
System model of the considered SM-MIMO-FD relay system.

We consider a single-user single-carrier SM-MIMO-FD relay system with a source node S, which transmits its signal to a destination node D via a relay node R as shown in Figure 1. Particularly, S and D operate in the half-duplex (HD) mode with $N_{S}$ transmitting antennas and $N_{D}$ receiving antennas, respectively. The relay node operates in the FD mode with $N_{\mathrm{tR}}$ transmitting antennas and $N_{\mathrm{rR}}$ receiving  antennas.

At time slot *t*, the received signal at R can be calculated as

(1) $$\begin{array}{c} y_{R}\left(t\right)=\sqrt{P_{S}}h_{i}^{R}x_{S}\left(t\right)+\sqrt{P_{R}}h_{j}^{R}x_{R}\left(t\right)+z_{R}\left(t\right), \end{array}$$

where $h_{i}^{R}$ and $h_{j}^{R}$ are respectively the channel vector from the *i*th active antenna of S to $N_{\mathrm{rR}}$ receiving antennas of R and from the *j*th active antenna of R to all reception antennas of R. These channels are assumed to undergo flat Rayleigh fading, which can be modeled by independent and identically distributed complex Gaussian random variables with zero mean and unit variance. $x_{S}$ and $x_{R}$ are the transmitted signals at the *i*th antenna of S and the *j*th antenna of R, respectively; $P_{S}$ and $P_{R}$ are the average transmission power at S and R, respectively; $z_{R}$ is the noise vector whose elements follow a complex Gaussian distribution with variance $\sigma^{2}$.

At the FD relay, we assume that the transmitting and receiving antennas are both directional, thus there will be no direct link which causes the self-interference (SI) from the transmit to the receive antenna. This SI is mainly due to reflections caused by multipath propagation. We also assume that the system can use all SIC techniques in the three domains, that is, propagation, analog and digital, to remove the SI [19,20]. Specifically, R can use all available isolation techniques to suppress SI. It can use the cross-polarization transmission to isolate the transmitting and receiving antennas [19,21]. In the analog domain, thanks to the SI awareness of analog circuits, the transmitted signal via the transmit antenna is collected and then subtracted from the received signal. The RSI is then converted to the digital domain for further SIC via digital signal processing. As R knows its transmitted signal, it can subtract the SI from the received signal by using SI channel estimation [20,22]. Thanks to all these SIC techniques, the relay node can achieve up to 110 dB SI suppression [23]. Moreover, since the SI is canceled from the received signal in the analog and digital domain by reconstructing the SI signal, the RSI is in effect the resulted errors due to the imperfect reconstruction or more correctly, the imperfect SI channel estimation. Moreover, as the digital-domain cancellation is done after a quantization operation, RSI at the relay $r_{\mathrm{SI}}$ can be modeled using complex Gaussian random variable [4,17,20,23] with zero mean and variance of $\sigma_{\mathrm{RSI}}^{2}$, that is $\sigma_{\mathrm{RSI}}^{2}=\tilde{\Omega }P_{R}$ where $\tilde{\Omega }$ denotes the SIC capability at the relay. Therefore, the received signal at R can be rewritten from (1) as

(2) $$\begin{array}{c} y_{R}\left(t\right)=\sqrt{P_{S}}h_{i}^{R}x_{S}\left(t\right)+r_{\mathrm{SI}}\left(t\right)+z_{R}\left(t\right), \end{array}$$

and the received signal at the destination D is then given by

(3) $$\begin{array}{c} y_{D}\left(t\right)=\sqrt{P_{R}}h_{j}^{D}x_{R}\left(t\right)+z_{D}\left(t\right), \end{array}$$

where $h_{j}^{D}$ is the channel vector from the *j*th antenna of R to $N_{D}$ receiving antennas of D; $z_{D}$ is the noise vector at D. Both S and R are assumed to have the same $N_{t}$ transmitting antennas for the same expected spectral efficiency. In the SM system, to estimate the transmitted bits, the receiver needs to use joint ML detection for both the activated transmit antenna and the *M*-ary modulated symbols. This joint detector is computationally complex, especially for the SM system with a large number of antennas. Near-ML low-complexity detectors such as those summarized in Reference [24] or that for the index modulation in the frequency domain [25] are more favorable for practical implementation. In this paper, as we are interested in analyzing the effect of the RSI due to the FD mode on the system performance, we assume that the receivers of both R and D can perfectly estimate the transmitted antenna index of the respective transmitters for the ML detection.

From (2) and (3), we can calculate the instantaneous signal-to-interference-plus-noise-ratios (SINRs) of S−R and R−D links as follows

(4) $$\begin{array}{c} \gamma_{R}=\frac{P_{S}{∥h_{i}^{R}∥}^{2}}{\sigma_{\mathrm{RSI}}^{2}+\sigma^{2}}={∥h_{i}^{R}∥}^{2}{\bar{\gamma }}_{R}, \end{array}$$

(5) $$\begin{array}{c} \gamma_{D}=\frac{P_{R}{∥h_{j}^{D}∥}^{2}}{\sigma^{2}}={∥h_{j}^{D}∥}^{2}{\bar{\gamma }}_{D}, \end{array}$$

where ${\bar{\gamma }}_{R}=\frac{P_{S}}{\sigma_{\mathrm{RSI}}^{2}+\sigma^{2}}$ and ${\bar{\gamma }}_{D}=\frac{P_{R}}{\sigma^{2}}$ denote the average SINR at R and the average signal-to-noise-ratio (SNR) at D, respectively.

Since the relay node uses the DF protocol, the instantaneous end-to-end SINR of the considered system is defined as

(6) $$\begin{array}{c} \gamma_{e2e}=\min\left(\gamma_{R},\gamma_{D}\right). \end{array}$$

where $\gamma_{R}$ and $\gamma_{D}$ are respectively the instantaneous SINRs at R and D.

## 3. Optimal Power Allocation for FD Mode

To find the optimal power allocation for the FD relay, we first calculate the SEP of the considered system using the definition given in Reference [26] as follows

(7) $$\begin{array}{c} \mathrm{SEP}=aE\left\{Q\left(\sqrt{b\gamma_{e2e}}\right)\right\}=\frac{a}{\sqrt{2\pi }}\int_{0}^{\infty }F_{\gamma_{e2e}}\left(\frac{t^{2}}{b}\right)e^{-\frac{t^{2}}{2}}dt, \end{array}$$

where *a* and *b* are constants whose values depend on the modulation types, for example, a=1,b=2 for the binary phase-shift keying (BPSK) modulation [26]. The values of *a* and *b* are determined using Table 6.1 of Reference [26]; Q(x) is the Gaussian function; $\gamma_{e2e}$ is the instantaneous end-to-end SINR of the considered system which is determined in (6); $F_{\gamma_{e2e}}\left(.\right)$ is cumulative distribution function (CDF) of $\gamma_{e2e}$ [21,27]. After some mathematical manipulations, (7) becomes

(8) $$\begin{array}{c} \mathrm{SEP}=\frac{a\sqrt{b}}{2\sqrt{2\pi }}\int_{0}^{\infty }\frac{e^{-bx/2}}{\sqrt{x}}F_{\gamma_{e2e}}\left(x\right)dx. \end{array}$$

To obtain the closed-form expression of (8), we calculate the CDF, $F_{\gamma_{e2e}}\left(x\right)$, of the probability that the instantaneous end-to-end SINR falls below a defined threshold. Mathematically, $F_{\gamma_{e2e}}\left(x\right)$ is expressed as

(9) $$\begin{array}{cc} F_{\gamma_{e2e}}\left(x\right) & =\mathrm{Pr}\left\{\log_{2}\left(N_{t}\right)+\log_{2}\left(1+\gamma_{e2e}\right)<R\right\}\\ & =\mathrm{Pr}\left\{\gamma_{e2e}<2^{R-\log_{2}\left(N_{t}\right)}-1\right\}, \end{array}$$

where R is the minimum data transmission rate of the considered system; the term $\log_{2}\left(N_{t}\right)$ denotes the number of bits which is used for activating the transmit antenna at the transmitters of (S or R).

Using the probability law of two independent variables A and B [28], that is Pr{A∪B}=Pr{A}+Pr{B}−Pr{A}Pr{B}, we have

(10) $$\begin{array}{cc} F_{\gamma_{e2e}}\left(x\right)= & \mathrm{Pr}\left\{\gamma_{e2e}<2^{R-\log_{2}\left(N_{t}\right)}-1\right\}=\mathrm{Pr}\left\{\gamma_{e2e}<x\right\}\\ = & \mathrm{Pr}\left\{\gamma_{R}<x\right\}+\mathrm{Pr}\left\{\gamma_{D}<x\right\}\\ \; & -\mathrm{Pr}\left\{\gamma_{R}<x\right\}\mathrm{Pr}\left\{\gamma_{D}<x\right\}, \end{array}$$

where $x=2^{R-\log_{2}\left(N_{t}\right)}-1$.

To calculate $F_{\gamma_{e2e}}\left(x\right)$ in (10), we first start with the CDF and probability distribution function (PDF) of the channel gain which follows Rayleigh fading distribution, that is,

(11) $$\begin{array}{cc} F_{{\left|h\right|}^{2}}\left(x\right) & ={\mathrm{Pr}\{|h|}^{2}<x\}=1-\exp\left(-\frac{x}{\Omega }\right),x⩾0, \end{array}$$

(12) $$\begin{array}{cc} f_{{\left|h\right|}^{2}}\left(x\right) & =\frac{1}{\Omega }\exp\left(-\frac{x}{\Omega }\right),x⩾0, \end{array}$$

where $\Omega =E\{|h{|}^{2}\}$ is the average channel gain; E denotes the expectation operator. In this paper, for the ease of presentation, we choose Ω=1 for all channel gains.

Then, we apply (11) and (12) to compute the probability in (10) as

(13) $$\begin{array}{cc} \mathrm{Pr}\{\gamma_{R}<x\} & =\mathrm{Pr}\left\{∥h_{i}^{R}{∥}^{2}{\bar{\gamma }}_{R}<x\right\}\\ & =\mathrm{Pr}\left\{∥h_{i}^{R}{∥}^{2}<\frac{x}{{\bar{\gamma }}_{R}}\right\}. \end{array}$$

Based on the CDF of the summation of channel gains [20], this probability is calculated as

(14) $$\begin{array}{c} \mathrm{Pr}\left\{\gamma_{R}<x\right\}=1-e^{-\frac{x}{{\bar{\gamma }}_{R}}}\sum_{i=0}^{N_{\mathrm{rR}}-1}\frac{1}{i!}\frac{x^{i}}{{\bar{\gamma }}_{R}^{i}}. \end{array}$$

Using similar calculations, we can obtain $\mathrm{Pr}\{\gamma_{D}<x\}$ as

(15) $$\begin{array}{c} \mathrm{Pr}\left\{\gamma_{D}<x\right\}=1-e^{-\frac{x}{{\bar{\gamma }}_{D}}}\sum_{j=0}^{N_{D}-1}\frac{1}{j!}\frac{x^{j}}{{\bar{\gamma }}_{D}^{j}}. \end{array}$$

Finally, $F_{\gamma_{e2e}}\left(x\right)$ in (10) can be given by

(16) $$\begin{array}{c} F_{\gamma_{e2e}}\left(x\right)=1-e^{-\frac{x}{{\bar{\gamma }}_{R}}-\frac{x}{{\bar{\gamma }}_{D}}}\sum_{i=0}^{N_{\mathrm{rR}}-1}\sum_{j=0}^{N_{D}-1}\frac{1}{i!j!}\frac{x^{i+j}}{{\bar{\gamma }}_{R}^{i}{\bar{\gamma }}_{D}^{j}}. \end{array}$$

Remark: As shown in (16), the active antenna at the transmitter is hidden in the variable *x* because $x=2^{R-\log_{2}\left(N_{t}\right)}-1$. Moreover, under the assumption that the receiver can estimate the index of the transmitter’s activated antenna, it can successfully decode the transmitted bits used to modulate the active antenna. Substituting $F_{\gamma_{e2e}}\left(x\right)$ in (16) into (8), we obtain the closed-form expression of SEP as follows:

(17) $$\begin{array}{c} \begin{array}{cc} \mathrm{SEP}= & \frac{a\sqrt{b}}{2\sqrt{2\pi }}\left[\int_{0}^{\infty }\frac{e^{-bx/2}}{\sqrt{x}}dx-\int_{0}^{\infty }\frac{e^{-bx/2}}{\sqrt{x}}e^{-\frac{x}{{\bar{\gamma }}_{R}}-\frac{x}{{\bar{\gamma }}_{D}}}\sum_{i=0}^{N_{\mathrm{rR}}-1}\sum_{j=0}^{N_{D}-1}\frac{1}{i!j!}\frac{x^{i+j}}{{\bar{\gamma }}_{R}^{i}{\bar{\gamma }}_{D}^{j}}dx\right]\\ = & \frac{a}{2}-\frac{a\sqrt{b}}{2\sqrt{2\pi }}\sum_{i=0}^{N_{\mathrm{rR}}-1}\sum_{j=0}^{N_{D}-1}\frac{\Gamma (i+j+\frac{1}{2})}{i!j!{\bar{\gamma }}_{R}^{i}{\bar{\gamma }}_{D}^{j}{\left(\frac{1}{{\bar{\gamma }}_{R}}+\frac{1}{{\bar{\gamma }}_{D}}+\frac{b}{2}\right)}^{i+j+\frac{1}{2}}}\cdot \end{array} \end{array}$$

It is worth noting that we have used equations ([29] (3.361.2)) and ([29] (3.381.4))to solve the first and second integrals in (17), respectively.

For the purpose of improving system performance and reducing the impact of the RSI in FD mode, we can calculate the optimal transmission power of R to minimize the system SEP. The optimal transmission power of R for minimizing the system SEP, denoted by $P_{R}^{*}$, is defined as

(18) $$\begin{array}{c} P_{R}^{*}=\arg\;\min_{P_{R}}\mathrm{SEP}. \end{array}$$

To explicitly determine the minSEP in (18), we begin with two terms in (17), that is, ${\mathrm{SEP}}_{1}$ and ${\mathrm{SEP}}_{2}$, as follows

(19) $$\begin{array}{c} \mathrm{SEP}=\underset{{\mathrm{SEP}}_{1}}{\underset{︸}{\frac{a}{2}}}-\underset{{\mathrm{SEP}}_{2}}{\underset{︸}{\frac{a\sqrt{b}}{2\sqrt{2\pi }}\sum_{i=0}^{N_{\mathrm{rR}}-1}\sum_{j=0}^{N_{D}-1}\frac{\Gamma (i+j+\frac{1}{2})}{i!j!{\bar{\gamma }}_{R}^{i}{\bar{\gamma }}_{D}^{j}{\left(\frac{1}{{\bar{\gamma }}_{R}}+\frac{1}{{\bar{\gamma }}_{D}}+\frac{b}{2}\right)}^{i+j+\frac{1}{2}}}}}, \end{array}$$

where

(20) $$\begin{array}{c} {\mathrm{SEP}}_{1}=\frac{a}{2}, \end{array}$$

and

(21) $$\begin{array}{c} {\mathrm{SEP}}_{2}=\frac{a\sqrt{b}}{2\sqrt{2\pi }}\sum_{i=0}^{N_{\mathrm{rR}}-1}\sum_{j=0}^{N_{D}-1}\frac{\Gamma (i+j+\frac{1}{2})}{i!j!{\bar{\gamma }}_{R}^{i}{\bar{\gamma }}_{D}^{j}{\left(\frac{1}{{\bar{\gamma }}_{R}}+\frac{1}{{\bar{\gamma }}_{D}}+\frac{b}{2}\right)}^{i+j+\frac{1}{2}}}. \end{array}$$

As mentioned in Section 3, after (7), since *a* and *b* are constants, ${\mathrm{SEP}}_{1}=\frac{a}{2}$ is also constant. Therefore, we have

(22) $$\begin{array}{c} \min\mathrm{SEP}=\min\left({\mathrm{SEP}}_{1}-{\mathrm{SEP}}_{2}\right)={\mathrm{SEP}}_{1}-\max{\mathrm{SEP}}_{2}=\max{\mathrm{SEP}}_{2}. \end{array}$$

Next, (22) can be rewritten as

(23) $$\begin{array}{c} \min\mathrm{SEP}=\max{\mathrm{SEP}}_{2}=\max\left(\frac{a\sqrt{b}}{2\sqrt{2\pi }}\sum_{i=0}^{N_{\mathrm{rR}}-1}\sum_{j=0}^{N_{D}-1}\frac{\Gamma (i+j+\frac{1}{2})}{i!j!{\bar{\gamma }}_{R}^{i}{\bar{\gamma }}_{D}^{j}{\left(\frac{1}{{\bar{\gamma }}_{R}}+\frac{1}{{\bar{\gamma }}_{D}}+\frac{b}{2}\right)}^{i+j+\frac{1}{2}}}\right), \end{array}$$

and (23) can be presented as

(24) $$\begin{array}{c} \min\mathrm{SEP}=\max\left(\frac{a\sqrt{b}}{2\sqrt{2\pi }}\sum_{i=0}^{N_{\mathrm{rR}}-1}\sum_{j=0}^{N_{D}-1}\frac{\Gamma (i+j+\frac{1}{2})}{i!j!}\times \frac{1}{{\bar{\gamma }}_{R}^{i}{\bar{\gamma }}_{D}^{j}{\left(\frac{1}{{\bar{\gamma }}_{R}}+\frac{1}{{\bar{\gamma }}_{D}}+\frac{b}{2}\right)}^{i+j+\frac{1}{2}}}\right). \end{array}$$

It is noted that, in (24), *a*, *b*, $N_{\mathrm{rR}}$ and $N_{D}$ are constants; *i* and *j* are antenna indices which do not depend on the transmission power of S and R. Moreover, $\Gamma (i+j+\frac{1}{2})$ is also a constant for the certain values of *i* and *j*. Therefore, (24) is maximized when

(25) $$\begin{array}{c} \frac{1}{{\bar{\gamma }}_{R}^{i}{\bar{\gamma }}_{D}^{j}{\left(\frac{1}{{\bar{\gamma }}_{R}}+\frac{1}{{\bar{\gamma }}_{D}}+\frac{b}{2}\right)}^{i+j+\frac{1}{2}}} \end{array}$$

is maximized or

(26) $$\begin{array}{c} {\bar{\gamma }}_{R}^{i}{\bar{\gamma }}_{D}^{j}{\left(\frac{1}{{\bar{\gamma }}_{R}}+\frac{1}{{\bar{\gamma }}_{D}}+\frac{b}{2}\right)}^{i+j+\frac{1}{2}} \end{array}$$

is minimized.

In summary, the minSEP in (18) is given by

(27) $$\begin{array}{cc} \min\mathrm{SEP} & =\min\left(\frac{a}{2}-\frac{a\sqrt{b}}{2\sqrt{2\pi }}\sum_{i=0}^{N_{\mathrm{rR}}-1}\sum_{j=0}^{N_{D}-1}\frac{\Gamma (i+j+\frac{1}{2})}{i!j!{\bar{\gamma }}_{R}^{i}{\bar{\gamma }}_{D}^{j}{\left(\frac{1}{{\bar{\gamma }}_{R}}+\frac{1}{{\bar{\gamma }}_{D}}+\frac{b}{2}\right)}^{i+j+\frac{1}{2}}}\right)\\ \; & =\min\left({\bar{\gamma }}_{R}^{i}{\bar{\gamma }}_{D}^{j}{\left(\frac{1}{{\bar{\gamma }}_{R}}+\frac{1}{{\bar{\gamma }}_{D}}+\frac{b}{2}\right)}^{i+j+\frac{1}{2}}\right). \end{array}$$

Denote $P_{R}=\alpha P_{S}$ and $f\left(\alpha \right)={\bar{\gamma }}_{R}^{i}{\bar{\gamma }}_{D}^{j}{\left(\frac{1}{{\bar{\gamma }}_{R}}+\frac{1}{{\bar{\gamma }}_{D}}+\frac{b}{2}\right)}^{i+j+\frac{1}{2}}$. Now we need to find $\alpha^{*}$, which is the optimal value of α. Then for this given $\alpha^{*}$, we can obtain $P_{R}^{*}$. The procedure for obtaining $\alpha^{*}$ and $P_{R}^{*}$ is summarized in the following Algorithm 1.

**Algorithm 1** Calculation of optimal $\alpha^{*}$ and $P_{R}^{*}$

1:Solve $\frac{\partial f(\alpha )}{\partial \alpha }=0$ for $\alpha =\alpha_{0}$;  2:**if**$\left\{\begin{array}{c} \alpha_{0}>0\\ \frac{\partial f(\alpha )}{\partial \alpha }<0\;\mathrm{for}\;\alpha <\alpha_{0}\\ \frac{\partial f(\alpha )}{\partial \alpha }>0\;\mathrm{for}\;\alpha >\alpha_{0} \end{array}\right.$  3:**then**  4:  Output optimal value of α  $\alpha^{*}=\alpha_{0}$;  thus  $P_{R}^{*}=\alpha^{*}P_{S}$  5:
**else**
6:    Output optimal value $\alpha^{*}=\emptyset$;  7:
**end**



We will explain step-by-step the process of Algorithm 1 as follows.

- **Step 1:** We take the derivative of $\frac{\partial f(\alpha )}{\partial \alpha }$ with respect to α and solve $\frac{\partial f(\alpha )}{\partial \alpha }=0$ to obtain the stationary point $\alpha_{0}$. Specifically, after some basic algebra calculations, we obtain the following equation
  (28) $$\begin{array}{c} f^{\prime }\left(\alpha \right)\leq 2\tilde{\Omega }P_{S}\alpha^{2}-2bP_{S}\alpha -9. \end{array}$$
  Then, $\alpha_{0}$ can be calculated as
  (29) $$\begin{array}{c} \alpha_{0}=\frac{bP_{S}+\sqrt{P_{S}\left(b^{2}P_{S}+18\tilde{\Omega }\right)}}{2\tilde{\Omega }P_{S}}. \end{array}$$
- **Step 2:** We check whether $\frac{\partial f(\alpha )}{\partial \alpha }$ is negative or positive in a specific interval to determine maximum or minimum point. If $\frac{\partial f(\alpha )}{\partial \alpha }$ is negative when $\alpha <\alpha_{0}$ and positive when $\alpha >\alpha_{0}$, an optimal transmission power $P_{R}^{*}$ exits and it is given by
  (30) $$\begin{array}{c} P_{R}^{*}=\frac{bP_{S}+\sqrt{P_{S}\left(b^{2}P_{S}+18\tilde{\Omega }\right)}}{2\tilde{\Omega }}. \end{array}$$
  It is worth noting that (30) is the bounded optimal transmission power at the FD relay node and it is often used for the systems with complex mathematical expressions.
- **Step 3:** Otherwise, if $\alpha^{*}=\emptyset$, depending on whether $\frac{\partial f(\alpha )}{\partial \alpha }$ is less or greater than 0, we can select an appropriate value of $\alpha^{*}$ to get $P_{R}^{*}$.

## 4. Numerical Results

In this section, to validate the derived mathematical expressions in the previous sections, we provide analytical results together with the Monte-Carlo simulation results for comparison. For ease of presentation, both S and R use two transmitting antennas, that is $N_{t}=2$, while the number of receiving antennas $N_{\mathrm{rR}}$ and $N_{D}$ are set to be equal and varies from 2 to 4 for performance evaluations. The SIC capability used for evaluation is $\tilde{\Omega }=\left\{-10,-5,0\right\}\;\mathrm{dB}$. For an LTE relay, the typical transmission power ranges from 23 dBm to 30 dBm. Taking 30 dBm for consideration, the RSI levels are $\sigma_{\mathrm{RSI}}^{2}=\left\{20,25,30\right\}$ dBm, respectively. In all figures, we define the average SNR for the case without optimization as follows: $\mathrm{SNR}=\frac{P_{S}}{\sigma^{2}}=\frac{P_{R}}{\sigma^{2}}$. In the case with optimization, the average SNR is defined by SNR at R, that is, $\mathrm{SNR}=\frac{P_{S}}{\sigma^{2}}$. The analytical curves are plotted using Equation (17) while the markers refer to Monte-Carlo simulation results. The simulation results were obtained using $10^{6}$ channel realizations.

**Figure 2:**
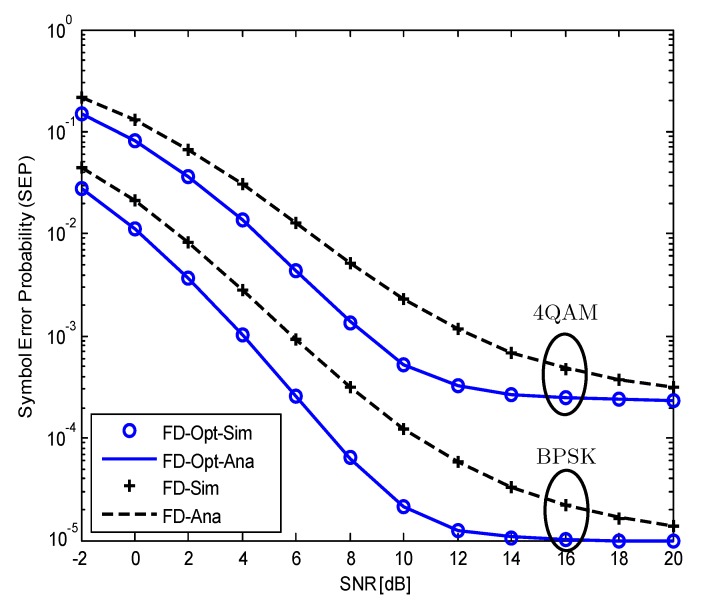
The SEP of the considered SM-MIMO-FD relay system with and without optimal power allocation versus the SNR for different modulation schemes, $N_{\mathrm{rR}}=N_{D}=4$.

Figure 2 plots the SEP of the considered SM-MIMO-FD relay system versus the SNR in dB for two modulation schemes, that is, BPSK (a=1, b=2) and 4-QAM (a=2, b=1). We should remind that *a* and *b* are constants, which depend on the types modulation scheme. These values for each type of modulation are given in Table 6.1 of Reference [26]. As shown in Figure 2, similar patterns of SEPs can be observed in both BPSK and 4-QAM modulation schemes. However, the system with 4-QAM modulation has higher SEP than the system with BPSK modulation. Moreover, the benefit of optimization is also reduced as the modulation order increases. Therefore, although our analysis method can be applied for all modulation types, we use the BPSK modulation in the following figures to clearly show the advantage of our proposed optimization algorithm in reducing the SEP of the considered SM-MIMO-FD relay system.

**Figure 3:**
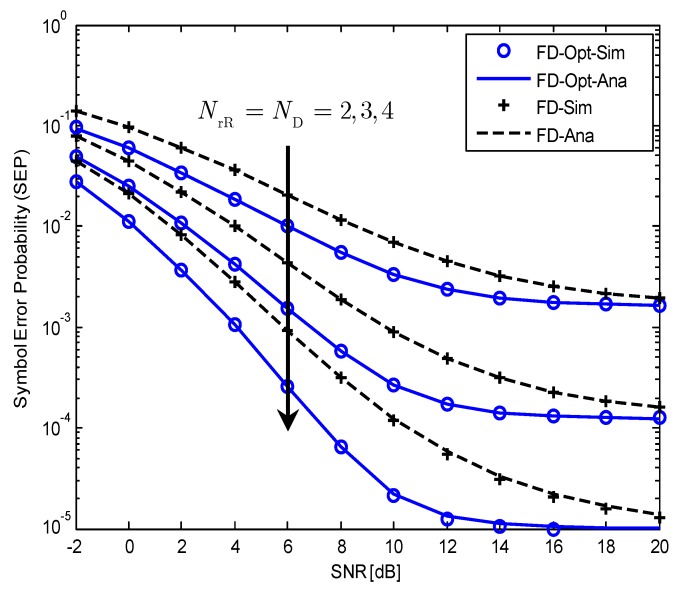
The SEP of the considered SM-MIMO-FD relay system with and without optimal power allocation when BPSK modulation is used, $N_{\mathrm{rR}}=N_{D}=2,3,4;\tilde{\Omega }=-10\;\mathrm{dB}$.

Figure 3 compares the SEPs of the SM-MIMO-FD relay system in two cases, that is α=1 (without optimal power allocation, $P_{R}=P_{S}$) and $\alpha =\alpha^{*}$ (with optimal power allocation, $P_{R}=P_{R}^{*}$) in (30) for different numbers of receiving antennas of R and D. Although we have used $N_{\mathrm{rR}}=N_{D}=2,3,4$ to obtain this figure, it is worth noting that we can use different numbers of receiving antennas at R and D for numerical calculation using the closed-form expression of SEP. The modulation used for evaluation is BPSK with parameters a=1,b=2. The typical SIC capability of $\tilde{\Omega }=-10\;\mathrm{dB}$ is used for calculation. It is easy to see from the figure that the analytical results perfectly match the simulation ones. Although the SEPs of both the cases with and without optimal power allocation suffer the same error floors in high SNR regime, the SEP with optimal power allocation is significantly lower than that without optimal power allocation in low SNR regime. For example, when SNR=8dB, the SEP in the case with $\alpha^{*}$ is approximately ten times less than the case without $\alpha^{*}$.

**Figure 4:**
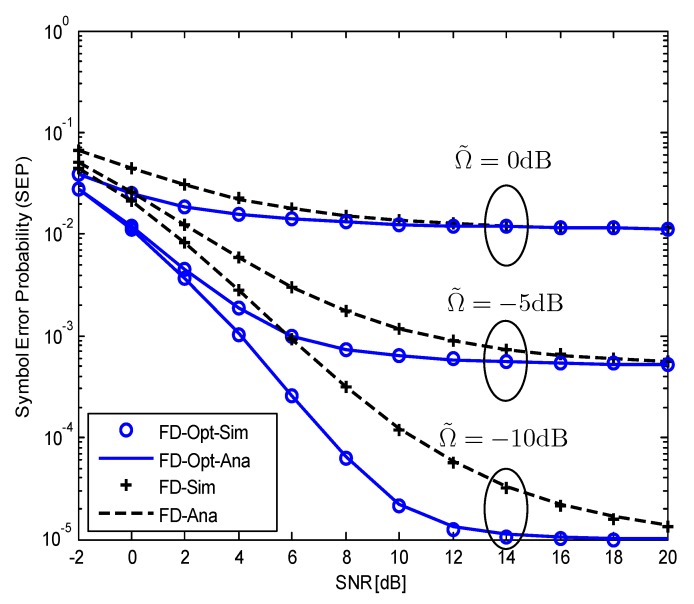
The impact of RSI on the SEP of the considered SM-MIMO-FD relay system with and without optimal power allocation; $\tilde{\Omega }=-10,-5,0\;\mathrm{dB};N_{\mathrm{rR}}=N_{D}=4$.

Figure 4 shows the impact of the RSI on the SEP of the system with and without optimal power allocation for different SIC capabilities, that is, $\tilde{\Omega }=-10,-5,0\;\mathrm{dB}$. When the RSI is small ($\tilde{\Omega }=-10\;\mathrm{dB}$), the difference in SEPs in the cases with and without using $\alpha^{*}$ is large. However, when the RSI becomes larger ($\tilde{\Omega }=-5\;\mathrm{dB}$), the benefit of using $\alpha^{*}$ decreases. Therefore, it is necessary to combine SIC techniques with optimal power allocation to achieve the best performance for this system. Figure 3 and Figure 4 can be served as the guideline to determine when the optimal power allocation should be used. Specifically, for SNR<20dB, we use $P_{R}^{*}$ to improve the system performance. For higher SNR, that is SNR>20dB, we use $P_{R}=P_{S}$ to reduce the signal processing complexity at the FD relay. The advantage of the system with optimization is that it requires only SNR≥8dB to achieve a reliable voice transmission.

## 5. Conclusions

In this paper, we have analyzed the performance of the SM-MIMO-FD DF relay system and derived the closed-form expression of the system SEP. Understanding the importance of the optimal power allocation to the SM-MIMO-FD relay system, especially in the case of imperfect SIC, we have proposed an optimal power allocation algorithm for the FD relay to minimize the system SEP. Both numerical and simulation results showed that the RSI has a substantial impact on the SEP performance. However, the SEP of the system with optimal power allocation is significantly lower compared with that of the system without optimal power allocation. This result confirms the effectiveness of using optimal power allocation for compensating the impact of the RSI in the SM-MIMO-FD DF relay system.
